# Determinants of tuberculosis among adult people living with HIV on antiretroviral therapy at public hospitals in Hawassa City, South Ethiopia

**DOI:** 10.3389/fepid.2024.1353760

**Published:** 2024-04-04

**Authors:** Ahmed Asefa, Habte Bolka, Endrias Markos Woldesemayat

**Affiliations:** College of Medicine and Health Sciences, Hawassa University, Hawassa, Ethiopia

**Keywords:** risk factors, tuberculosis, HIV-positive, adults, ART

## Abstract

**Background:**

The burden of tuberculosis (TB)/HIV co-infection is high in sub-Saharan African countries. The aim of the present study was to identify determinants of TB among people living with HIV (PLHIV) on antiretroviral therapy (ART) at public hospitals in Hawassa City Administration, Sidama Region, Ethiopia.

**Methods:**

A facility-based case-control study was conducted between 30 March and 30 April 2023. We employed a systematic random sampling to recruit participants. The cases were all adult PLHIV who developed TB after ART initiation, and the group without TB were all adult PLHIV who did not develop TB after their ART initiation. Data were collected from patients’ medical records using Kobo-tool and then exported to SPSS Version 26 for analysis. A multivariable logistic regression was used to identify the predictors of TB. Statistical significance was defined using the 95% confidence interval (CI).

**Result:**

A total of 124 cases and 249 people without TB participated in the study. In a multivariable logistic regression analysis, we identified five independent determinants of TB. These include age (adjusted odds ratio (AOR) = 2.7; 95% CI 1.4–5.2), patients’ residency (AOR = 6.4; 95% CI 2.8–14.5), WHO clinical stage III or IV (AOR = 6.7; 95% CI 3.2–14.0), isoniazid plus rifapentine (3HP) prophylaxis using (AOR = 0.5; 95% CI 0.2–0.9), and having other opportunistic infections (AOR = 3.6; 95% CI 1.7–7.6).

**Conclusion and recommendation:**

Several risk factors for TB were identified among PLHIV. Strengthening TB screening in advanced disease conditions, encouraging use of 3HP prophylaxis, and early diagnosis and treatment of opportunistic infections were recommended to reduce the incidence of TB among PLHIV.

## Background

Tuberculosis (TB) is the first opportunistic infection in the majority of people living with HIV (PLHIV) (1). It is one of the most common causes of death among PLHIV (2). HIV weakens the immune system, increases the vulnerability of being infected with TB, and is a major contributing factor to the progression from latent to active TB as well as a reason for changing the clinical manifestation of TB (3). The risk of developing TB is 15–21 times higher among PLHIV (4). TB, on the other hand, accelerates the progression and replication of HIV through the immune activation process, resulting in an increased viral load and a faster progression of the stages from HIV to AIDS (3). TB/HIV co-infections promote the severity of symptoms and increases the likelihood of fatality compared to HIV infection alone (5, 6).

A systematic review reported that the prevalence of TB/HIV co-infection was 25.59% (7). Globally, in 2021, there were 703,000 new cases of TB among PLHIV and Africa accounts for 69% of this incidence (8). The global trend in deaths from TB among PLHIV has increased from 209,000 in 2019 to 214,000 in 2020. The death rate in 2020 increased by 5.6% from the 2019 report (2). Globally, TB accounted for approximately one-third of AIDS-related deaths and was also the main cause of death among PLHIV (4, 9). TB/HIV co-infections usually need complex treatment regimens for longer treatment periods and are associated with poor adherence to treatment due to pill burden, higher rates of treatment failure, stigma and discrimination, and a high mortality rate (10, 11).

Ethiopia is one of the countries with the highest TB/HIV burden (7). The Federal Ministry of Health (FMOH) Ethiopia set the “End TB” strategy and other strategies to halt the transmission of TB, the Sustainable Development Goals, and local TB prevention approaches to decrease the incidence and death rate of TB (12). Early screening for TB and providing preventive TB preventative treatment and co-trimoxazole prophylaxis therapy (CPT) were interventions being implemented to reduce the incidence of TB among PLHIV in the country (13). However, the incidence of TB among PLHIV remained one of the public health problems in Ethiopia.

In 2020, the FMOH recommended starting a new antiretroviral medication, dolutegravir (DTG), in Ethiopia after the World Health Organization (WHO) recommendation in 2019 (14). It is a newly recommended medication regimen for PLHIV, including adults, adolescents, and pregnant women, due to its virologic efficacy, favorable tolerability, and toxicity profiles. To the best of our knowledge, there are no studies in Ethiopia that assessed the determinants of TB among PLHIV after the implementation of the new antiretroviral therapy (ART) regimen. The aim of the present study was to identify the determinants of TB among adult PLHIV on ART at public hospitals in Hawassa City, Sidama Region, Ethiopia.

## Methods

### Study setting

The present study was conducted at Hawassa University Comprehensive Specialized Hospital (HUCSH) and Adare General Hospital in Hawassa City, Sidama Region, Ethiopia, with a population of approximately 400,000 people. There are four public hospitals and 10 health centers in the Hawassa City Administration. HUCSH is a public comprehensive specialized referral hospital in the region; it provides service for approximately 18–20 million people in the South Ethiopia and Oromia Regions. The other hospital included in our study was Adare Generalized Hospital, which has approximately 21 different service units and an ART clinic, and the hospital served approximately 2,386 ART followers at the time we conducted the study.

### Population

All adult PLHIV aged at least 18 years and who were receiving care between 30 April 2020 and 30 April 2023 were considered to be our source population. All randomly selected adult PLHIV aged at least 18 years and who were on ART at selected hospitals during the study period were our study population. All adult PLHIV who developed TB after the initiation of ART and were diagnosed by either clinical or laboratory means during the study period were considered to be our cases. People without TB were all PLHIV in the hospitals who did not develop any form of TB after the initiation of ART before or during the period of 30 April 2020 to 30 April 2023. All PLHIV aged at least 18 years in the selected hospitals and who initiated ART during the study period were included in the study; those who started the follow-up before and after the aforementioned period and whose medical records were incomplete were excluded from the study.

### Sample size determination

A sample size calculation was carried out using a double population proportion formula considering the assumptions of power of 80%, the case-to-people without TB ratio of 1:2, and type I error of 5%. The sample size was calculated using Epi Info version 7.2.5.0 software. Having diabetes mellitus was used as the predictor variable that gave us a high sample size (15), which was 113 cases and 226 individuals without TB; adding 10% for non-response, the total sample size was 373 (124 cases and 249 individuals without TB). The sample size calculation is described further in the Supplementary Table S1.

### Sampling

There are four public hospitals that provide ART services in Hawassa City Administration. Of them, two hospitals—Adare General Hospital and HUCSH—were purposively selected because they had high numbers of PLHIV. The identification of cases and people without TB was done by the principal investigator. Based on the eligibility criteria, a list of cases and people without TB was prepared using a unique identification number from ART clinic records, and then a proportional allocation was done for the facilities. Systematic sampling techniques were used to select cases and people without TB. PLHIV on ART who fulfilled the inclusion criteria were included in the study, with a case to people without TB ratio of 1:2. Finally, 124 TB/HIV-infected patients were selected as cases, and 249 as HIV-infected people without TB.

### Variables of the study

The dependent variable considered in the study was the occurrence of TB among HIV-positive adults on ART. The independent variables that were measured in the study were sociodemographic characteristics, such as gender, age, marital status, educational status, occupation, residence, and family size. The clinical factors included in the study were opportunistic infection, WHO clinical stage, CD4 level, viral load, body mass index (BMI), hemoglobin level, time on ART before diagnosis of TB, isoniazid plus rifapentine (3HP) prophylaxis, CPT prophylaxis, and functional status of the study participant.

### Operational definitions

TB occurrence refers to HIV-positive adults who developed any form of TB after the initiation of ART as determined by clinical or laboratory methods between 20 April 2020 and 30 April 2023 at the selected hospitals in Hawassa City.

Adherence to ART was defined as follows: a score in the range of 0–5 was considered poor adherence; a score of 6–7 was considered fair adherence; and a score of 8 was considered good adherence. These scores were taken directly from the patient ART registry (16).

The functional status of the patient was categorized as working if the patient could perform his/her usual work in or out of the house, ambulatory if he/she could perform activities of daily living, and bedridden if he/she was not able to perform activities of daily living (17).

### Data collection tool and procedure

The data were collected using an electronic data abstraction checklist on a smart phone (KOBO Toolbox). The data abstraction format was prepared by reviewing the ART register, HIV care/follow-up charts, and TB treatment register and patients’ medical records. We collected data on sociodemographic variables, behavioral factors, and clinical factors. Four experienced nurses on ART service participated in the data collection.

### Data quality control

Training was delivered for the data collectors on the purpose of the study, the data collection tool, and KOBO-collect Toolbox. Data collectors sent the electronic data to the principal investigator, who owned the KOBO server. The principal investigator checked the collected data for completeness, accuracy, and consistency throughout the data collection period and provided feedback to the data collectors. Supervision was carried out by the principal investigator.

### Data processing and analysis

The collected data were exported from KOBO Toolbox and then imported into SPSS version 26 statistical software, in which we conducted the analysis. Data completeness and consistency were checked by running frequencies of each variable and have been corrected. Cross-tabulation was performed to compare the distribution of each independent variable with the outcome variable. Bivariate logistic regression analysis was used to select candidate variables for multivariate logistic regression analysis. Variables with *p*-values <0.25 in the binary model were entered into the multivariate logistic regression model in order to control the effect of different confounding factors. The adjusted odds ratio (AOR) along with the 95% confidence interval (CI) was used to determine the strength of association and a *p*-value <0.05 was considered to be statistically significant. The Hosmer–Lemeshow test was used to check the model’s goodness of fit.

### Ethics

Ethical clearance was obtained from the institutional review board (IRB) of Hawassa University, College of Medicine and Health Sciences (Ref. No: IRB/277/15 on 30 March 2023). A letter of support was obtained from the school of Public Health, Hawassa University. HUCSH and AGH provided permission to conduct the study. Data retrieved were kept anonymously. Except for the investigators, no one had access to the anonymous data set.

## Results

### Sociodemographic characteristics

In this study, a total of 124 cases and 249 people without TB were included. High proportions of women were involved in both groups: 86 (69.4%) cases and 158 (63.5%) people without TB. The median age was 47 years [interquartile range (IQR) 40–56] and 40 years (IQR 32.5–51) for cases and people without TB, respectively. The mean ± SD BMI of the cases was 19.4 ± 1.7 kg/m^2^ and that of the people without TB was 19.8 ± 1.9 kg/m^2^. The majority of participants were married (62.9% of cases and 48.6% of people without TB). A high proportion of participants came from urban areas (91.1% of cases and 62.7% of people without TB). In total, 174 participants (66 cases and 108 people without TB) attended at least secondary school. Employed participants constituted 67 (51.6%) cases and 155 (55.4%) people without TB. The family size for 99 cases and 214 people without TB was less than five (Table 1).

**Table 1:** Sociodemographic characteristics of PLHIV at public hospitals in Hawassa City, Sidama Region, Southern Ethiopia.

Characteristics	Categories	Case		Control	
		Number	Percent	Number	Percent
Gender	Male	38	30.6	91	36.5
	Female	86	69.4	158	63.5
Age in years	18–39	36	29	132	53
	≥40	88	71	117	47
Residency	Rural	11	8.9	93	37.3
	Urban	113	91.1	156	62.7
Body mass index	<18.5	26	21	46	18.5
	≥18.5	98	79	203	81.5
Marital status	Single	13	10.5	48	18.5
	Married	78	62.9	121	48.6
	Others^a^	33	26.6	82	32.9
Educational status	No education	16	12.9	37	14.2
	Primary school	32	25.8	74	29.7
	Secondary and above	76	61.3	138	55.4
Occupational status	Unemployed	60	48.4	111	44.6
	Employed	67	51.6	155	55.4
House hold size	1–4	99	79.8	214	85.9
	≥5	25	20.2	35	14.1

^a^ Others: divorce or widowed.

### Behavioral and clinical characteristics

The majority of the cases (57, 46%) and people without TB (143, 53.7%) received TDF + 3TC + DTG combination ART. A high proportion of cases were at WHO clinical stage III/IV (144, 70.9%). On the contrary, only 41 (17.7%) people without TB were at WHO clinical stage III/IV. The majority of cases (64, 53.3%) and people without TB (115, 46.6%) had a CD4 count of 200–499 cell/ml. More than half of the cases (67, 54%) and people without TB (141, 56.6%) had good adherence to ART. Time using ART was >5 years for 73 (58.9%) cases and 145 (58.2%) people without TB. In total, 86 (69.4%) cases and 186 (74.7%) people without TB were working. The majority of respondents (103 (83.1%) cases and 152 (69%) people without TB) had a history of opportunistic infections (Table 2).

**Table 2:** Behavioral and clinical characteristics of PLHIV at public hospitals in Hawassa City, Sidama Region, Southern Ethiopia.

Characteristics	Categories	Case		Control	
		Number	Percent	Number	Percent
ART regimen	1d	5	4	19	7.7
	1e	43	34.7	70	28.2
	1j	57	46	143	53.7
	2h	11	8.9	7	2.8
	2i	8	6.5	9	3.6
WHO clinical stage	Stage I/II	32	25.8	205	82.3
	Stage III/IV	92	74.2	41	17.7
CD4 count (cells/ml)	≥500	29	24.2	107	43.3
	200–499	64	53.3	115	46.6
	<200	27	22.5	25	10.1
Viral load (copies/ml)	>1,000	27	23.9	49	22
	<1,000	86	76.1	174	78
3HP	Yes	100	81.3	170	68.5
	No	23	18.7	78	31.5
CPT	Yes	67	54	84	33.7
	No	57	46	165	66.3
Opportunistic infection	Yes	103	83.1	97	39
	No	21	16.9	152	69
Functional status	Working	86	69.4	186	74.7
	Ambulatory/bedridden	38	30.6	63	25
ART adherence	Good	67	54	141	56.6
	Fair	33	26.6	68	27.3
	Poor	24	19.4	40	16.1
Duration on ART	>5	73	58.9	145	58.2
	1–5	51	41.1	104	41.8

1d, AZT + 3TC + EFV; 1e, TDF + 3TC + EFV; 1j, TDF + 3TC + DTG; 2i, ABC + 3TC + LVP/r; 2h, TDF + 3TC + ATV/r.

### Risk factors for TB among HIV-positive adults on ART

In a bivariate logistic regression analysis, variables like age, place of residency, marital status, WHO clinical staging, CD4 count, isoniazid plus rifapentine prophylaxis, CPT, and other opportunistic infections were associated with the occurrence of TB. However, in a multivariable logistic regression analysis, five variables were found to be significantly associated with the occurrence of TB. The odds of TB among PLHIV who were aged over 40 years was 2.7 times higher than their counterparts (AOR = 2.7; 95% CI 1.4–5.2). The odds of TB among PLHIV who lived in urban areas was 6.4 times higher than among those who lived in rural areas (AOR = 6.4; 95% CI 2.8–14.5). The risk of TB among individuals with stage III/IV disease was 6.7 times higher than those with stage I/II disease (AOR = 6.7; 95% CI 3.2–14.0). The risk of TB among PLHIV who did not use 3HP prophylaxis was lower (AOR = 0.5; 95% CI 0.2–0.9). PLHIV with opportunistic infections had about four times the increased risk of TB (AOR = 3.6; 95% CI 1.7–7.6). Table 3 shows the risk factors of TB among PLHIV.

**Table 3:** Determinants of TB among PLHIV at public hospitals in Hawassa City, Sidama Region, Southern Ethiopia.

Variable	Categories	Case	Control	COR (95% CI)	AOR (95% CI)
Age	18–39	36	117		
	≥40	88	132	2.2 (1.6–3.4)	2.7 (1.4–5.2)
Residency	Rural	11	93		
	Urban	113	156	6.1 (3.1–12.0)	6.4 (2.8–14.5)
Marital status	Single	13	48		
	Married	33	121	0.6 (0.5–2.0)	0.6 (0.2–1.6)
	Others^a^	78	82	3.4 (1.7–6.7)	1.9 (0.8–4.5)
WHO clinical stage	I/II	32	205		
	III/IV	92	41	13.4 (8.0–22.5)	6.7 (3.2–14.0)
CD4 count	>500	29	29		
	200–499	64	64	2.1 (1.2–3.4)	1.3 (0.6–2.8)
	<200	27	27	4.0 (2.0–7.9)	1.9 (0.6–6.2)
3HP	Yes	100	170	0.5 (0.3–0.9)	0.5 (0.2–0.9)
	No	23	78		
CPT	Yes	67	84		
	No	57	165	0.4 (0.3–0.7)	1.4 (0.6–3.2)
Opportunistic infection	Yes	103	97	7.6 (4.5–13.1)	3.6 (1.7–7.6)
	No	21	152		

^a^ Others, Divorce or widowed.

## Discussion

TB and HIV remain major public health problems in several countries, including Ethiopia. Several sociodemographic, clinical, and behavioral risk factors have been identified as risk factors of TB among PLHIV enrolled in ART. In the present study, old age, being an urban resident, being in the advanced stages of HIV, and having opportunistic infections also increased the risk of TB, while the provision of isoniazid plus rifapentine prophylaxis was identified as a protective factor of TB among PLHIV.

Due to their movement from one place to another, there is an increased risk of TB among young adults. Reports from South Africa (18), South Sudan (19), northeastern Ethiopia (20), and Arba Minch in southern Ethiopia (21) revealed an increased risk of TB among younger PLHIV. Contrary to these reports, in the present study, elderly PLHIV had an increased risk of TB infection. Our results are in line with findings from Nigeria (22), southern Ethiopia (5), and Horro Guduru Wollega Zone in Oromia Region, Ethiopia (23). A progressive loss of physiologic reserves among the elderly may lead to increased vulnerability to mycobacterium infection and to the disease. We suggest that care should be given for elderly PLHIV to minimize the risk of TB developing among them.

Contrary to our finding, reports from Dessie (24), Addis Ababa (25), and Debre Markos Hospitals (26) found that place of residence was not statistically associated with the occurrence of TB. This might have happened by chance. The likelihood of having TB among urban residents was higher among participants in the present study. This finding is consistent with reports from other settings, such as Burkina Faso (27), Amhara Region (28), and Western Ethiopia (15). The reason for the increased risk of TB among urban PLHIV could be related to overcrowded living conditions and the increased mobility of urban dwellers; this increases the risk of transmission of TB in urban areas.

The other independent predictor of TB in the present study was the WHO clinical staging. PLHIV with WHO clinical stage III or IV had a higher risk of developing TB than those with WHO clinical stage I or II. This result is consistent with the findings in other studies. Reports from West Bengal, India (29), Germany (30), Nepal (31), Saudi Arabia (32), South Africa (33), Tanzania (34), southwest Ethiopia (35), Addis Ababa (25), and Dessie Referral Hospital (24) showed an increased risk of TB among PLHIV with advanced WHO clinical stages. With advanced clinical stages, the immunity of PLHIV reduces. Though TB can occur at any WHO clinical stage, it is more common in the advanced stages due to reduced patient immunity.

Our results also showed that PLHIV who developed other opportunistic infections had an increased risk of TB. Similar findings were reported in studies from Addis Ababa (25), Dessie (24), and Debre Birhan towns in Ethiopia (36). The occurrence of opportunistic infections indicates the presence of reduced immune function in the patient, which could favor the occurrence of TB. The National Strategy recommends chemoprophylaxis for PLHIV on ART to prevent the occurrence of opportunistic infections (3), which could minimize the risk of developing opportunistic infections, including TB. Our analysis identified PLHIV who took isoniazid plus rifapentine were more protected from TB. The WHO suggests taking a complete dose of 3HP, weekly isoniazid plus rifapentine (3HP) prophylaxis for 3 months for PLHIV without active TB, irrespective of their immune status or whether they are on ART. This can reduce the risk of TB among PLHIV (8).

Since secondary data were used in the present study, it was impossible to include factors such as monthly income and housing conditions like poor ventilation in the assessment. These factors might be important predictors of TB that we did not show in our results. In addition, variables such as behavioral factors, history of contact with known cases of TB, and blood hemoglobin level were incomplete for many of the participants in the study; therefore, we excluded these characteristics from our analysis. These issues could be considered the main limitations of the current study.

## Conclusion

This study showed that elderly PLHIV, urban residency, being in the advanced stages of HIV, and having opportunistic infections were identified as significant determinants for the occurrence of TB among the study participants. On the other hand, taking isoniazid plus rifapentine prophylaxis had a protective effect against the development of TB. Screening for TB, updating TB preventive prophylaxis in the new ART regimen, and implementing it may reduce the burden of TB among PLHIV. Attention should be given to elderly PLHIV, patients with advanced stages of HIV, strengthening isoniazid plus rifapentine prophylaxis among adult PLHIV, and the early screening, prevention, and management of opportunistic infections among PLHIV.

## Data Availability

The raw data supporting the conclusions of this article will be made available by the authors, without undue reservation.
