# Supplementary material for: Determinants of tuberculosis among adult people living with HIV on antiretroviral therapy at public hospitals in Hawassa City, South Ethiopia
Source: Front Epidemiol. 2024 Apr 4;4:1353760. doi: 10.3389/fepid.2024.1353760 (PMC11025533; doi:10.3389/fepid.2024.1353760)
Supplement: Supplementary file 1 [file Table1.docx]

**Table 1.** Socio-demographic characteristics of PLHIV at public hospitals in Hawassa City, Sidama Region, Southern Ethiopia

| **Characteristics** | **Categories** | **Case** | | **Control** | |
| --- | --- | --- | --- | --- | --- |
|  |  | **Number** | **Percent** | **Number** | **Percent** |
| Gender | Male | 38 | 30.6 | 91 | 36.5 |
|  | Female | 86 | 69.4 | 158 | 63.5 |
| Age in years | 18-39 | 36 | 29 | 132 | 53 |
|  | ≥40 | 88 | 71 | 117 | 47 |
| Residency | Rural | 11 | 8.9 | 93 | 37.3 |
|  | Urban | 113 | 91.1 | 156 | 62.7 |
| Body mass index | <18.5 | 26 | 21 | 46 | 18.5 |
|  | ≥18.5 | 98 | 79 | 203 | 81.5 |
| Marital status | Single | 13 | 10.5 | 48 | 18.5 |
|  | Married | 78 | 62.9 | 121 | 48.6 |
|  | Others | 33 | 26.6 | 82 | 32.9 |
| Educational status | No education | 16 | 12.9 | 37 | 14.2 |
|  | Primary school | 32 | 25.8 | 74 | 29.7 |
|  | Secondary and above | 76 | 61.3 | 138 | 55.4 |
| Occupational status | Unemployed | 60 | 48.4 | 111 | 44.6 |
|  | Employed | 67 | 51.6 | 155 | 55.4 |
| House hold size | 1-4 | 99 | 79.8 | 214 | 85.9 |
|  | ≥5 | 25 | 20.2 | 35 | 14.1 |

## Others, Divorce or widowed
