# Supplementary material for: Determinants of tuberculosis among adult people living with HIV on antiretroviral therapy at public hospitals in Hawassa City, South Ethiopia
Source: Front Epidemiol. 2024 Apr 4;4:1353760. doi: 10.3389/fepid.2024.1353760 (PMC11025533; doi:10.3389/fepid.2024.1353760)
Supplement: Supplementary file 2 [file Table2.docx]

**Table 2.** Behavioral and clinical characteristics of PLHIV at public hospitals in Hawassa City, Sidama Region, Southern Ethiopia

| **Characteristics** | **Categories** | **Case** | | **Control** | |
| --- | --- | --- | --- | --- | --- |
|  |  | **Number** | **Percent** | **Number** | **Percent** |
| ART regimen | 1d | 5 | 4 | 19 | 7.7 |
|  | 1e | 43 | 34.7 | 70 | 28.2 |
|  | 1j | 57 | 46 | 143 | 53.7 |
|  | 2h | 11 | 8.9 | 7 | 2.8 |
|  | 2i | 8 | 6.5 | 9 | 3.6 |
| WHO clinical stage | Stage I/ II | 32 | 25.8 | 205 | 82.3 |
|  | Stage III/IV | 92 | 74.2 | 41 | 17.7 |
| CD4 count | ≥500 cell/ml | 29 | 24.2 | 107 | 43.3 |
|  | 200-499 cell/ml | 64 | 53.3 | 115 | 46.6 |
|  | <200 cell/ml | 27 | 22.5 | 25 | 10.1 |
| Viral load | >1000 copies/ml | 27 | 23.9 | 49 | 22 |
|  | < 1000 copies/ml | 86 | 76.1 | 174 | 78 |
| 3HP | Yes | 100 | 81.3 | 170 | 68.5 |
|  | No | 23 | 18.7 | 78 | 31.5 |
| CPT | Yes | 67 | 54 | 84 | 33.7 |
|  | No | 57 | 46 | 165 | 66.3 |
| Opportunistic infection | Yes | 103 | 83.1 | 97 | 39 |
|  | No | 21 | 16.9 | 152 | 69 |
| Functional status | Working | 86 | 69.4 | 186 | 74.7 |
|  | Ambulatory/bedridden | 38 | 30.6 | 63 | 25 |
| ART adherence | Good | 67 | 54 | 141 | 56.6 |
|  | Fair | 33 | 26.6 | 68 | 27.3 |
|  | Poor | 24 | 19.4 | 40 | 16.1 |
| Duration on ART | >5 | 73 | 58.9 | 145 | 58.2 |
|  | 1-5 | 51 | 41.1 | 104 | 41.8 |

Abbreviations: CI= confidence interval, 1d=AZT+3TC+EFV, 1e=TDF+3TC+EFV, 1j=TDF+3TC+DTG, 2i=ABC+3TC+LVP/r, 2h=TDF+3TC+ATV/r, CPT=co-trimoxazole prophylaxis therapy; TB, tuberculosis; 3HP=isoniazid Plus rifapentine, SD=standard deviation
