# Supplementary material for: Determinants of tuberculosis among adult people living with HIV on antiretroviral therapy at public hospitals in Hawassa City, South Ethiopia
Source: Front Epidemiol. 2024 Apr 4;4:1353760. doi: 10.3389/fepid.2024.1353760 (PMC11025533; doi:10.3389/fepid.2024.1353760)
Supplement: Supplementary file 3 [file Table3.docx]

**Table 3.** Determinants of TB **among PLHIV** at public hospitals in Hawassa City, Sidama Region, Southern Ethiopia

| **Variable** | **Categories** | **Case** | **Control** | **COR (95%CI)** | **AOR (95%CI)** |
| --- | --- | --- | --- | --- | --- |
| Age | 18-39 | 36 | 117 |  |  |
|  | ≥40 | 88 | 132 | 2.2 (1.6-3.4) | 2.7 ( 1.4-5.2) |
| Residency | Rural | 11 | 93 |  |  |
|  | Urban | 113 | 156 | 6.1 (3.1-12.0) | 6.4 (2.8-14.5) |
| Marital status | Single | 13 | 48 |  |  |
|  | Married | 33 | 121 | 0.6 (0.5-2.0) | 0.6 (0.2-1.6) |
|  | Others* | 78 | 82 | 3.4 (1.7-6.7) | 1.9 (0.8-4.5) |
| WHO clinical stage | I/ II | 32 | 205 |  |  |
|  | III/IV | 92 | 41 | 13.4 (8.0-22.5) | 6.7 (3.2-14.0) |
| CD4 count | >500 | 29 | 29 |  |  |
|  | 200-499 | 64 | 64 | 2.1 (1.2-3.4) | 1.3 (0.6-2.8) |
|  | <200 | 27 | 27 | 4.0 (2.0-7.9) | 1.9 (0.6-6.2) |
| 3HP | Yes | 100 | 170 | 0.5 (0.3-0.9) | 0.5 (0.2-0.9) |
|  | No | 23 | 78 |  |  |
| CPT | Yes | 67 | 84 |  |  |
|  | No | 57 | 165 | 0.4 (0.3-0.7) | 1.4 (0.6-3.2) |
| Opportunistic infection | Yes | 103 | 97 | 7.6 (4.5-13.1) | 3.6 (1.7-7.6) |
|  | No | 21 | 152 |  |  |

Abbreviations: BMI=body mass index; CI= confidence interval, CPT=co-trimoxazole prophylaxis therapy; TB, tuberculosis; 3HP=isoniazid Plus rifapentine, SD, standard deviation; *Others, Divorce or widowed
