# Supplementary material for: Determinants of tuberculosis among adult people living with HIV on antiretroviral therapy at public hospitals in Hawassa City, South Ethiopia
Source: Front Epidemiol. 2024 Apr 4;4:1353760. doi: 10.3389/fepid.2024.1353760 (PMC11025533; doi:10.3389/fepid.2024.1353760)
Supplement: Supplementary file 4 [file Table4.docx]

**Supplementary table 4.** Assumptions used for sample size calculation

| **Main factor** | **CI (%)** | **Power (%)** | **Case to group without TB ratio** | **% of exposure group without TB** | **AOR** | **Sample size** | | |
| --- | --- | --- | --- | --- | --- | --- | --- | --- |
|  |  |  |  |  |  | Case | Group without TB | Total with the 10% non-response |
| Aged ≥35 years [^16^](#_ENREF_16) | 95 | 80 | 1:2 | 47.5 | 2.63 | 67 | 133 | 200 |
| previous TB history [^17^](#_ENREF_17) | 95 | 80 | 1:2 | 37.4 | 2.41 | 76 | 152 | 228 |
| having diabetic mellitus [^15^](#_ENREF_15) | 95 | 80 | 1:2 | 4.2 | 3.63 | 124 | 249 | 373 |
| WHO stage III $ IV [^15^](#_ENREF_15) | 95 | 80 | 1:2 | 45.8 | 2.29 | 87 | 173 | 260 |
| CD IV level < 200 cell/ml [^17^](#_ENREF_17) | 95 | 80 | 1:2 | 41.9 | 2.02 | 116 | 151 | 347 |
